# A hybrid case of eosinophilic folliculitis and eosinophilic cellulitis associated with hypereosinophilic syndrome

**DOI:** 10.1002/ski2.407

**Published:** 2024-06-16

**Authors:** Domniki Iatropoulou, Cathryn Sprenger, Richard Griffiths, Nilukshi Wijesuriya, Bernard Ho

**Affiliations:** ^1^ Department of Dermatology St George's University Hospitals NHS Foundation Trust London UK; ^2^ Department of Pathology St George's Hospital NHS Foundation Trust London UK

## Abstract

Eosinophilic cellulitis or Wells syndrome encompasses distinct histopathological features but can also be associated with eosinophilic related conditions like hyper eosinophilic syndrome (HES) or eosinophilic granulomatosis with polyangiitis (EGPA) (Churg–Strauss syndrome). We report a case of a Turkish 41‐year‐old female who presented in clinic with pruritus and tenderness on her chest and breasts, having received several courses of antibiotics for recurrent abscess formation. A year before she had been diagnosed with HES with multiorgan involvement that included biopsy proven eosinophilic folliculitis, and prompted further investigation including bone marrow aspiration that revealed T cell clonality. Biopsy of her rash revealed eosinophilic infiltration of the dermis with flame figures. Ongoing respiratory symptoms and a history of childhood asthma were suggestive of EGPA. This case highlights important associations that should be considered in the investigation of Wells syndrome.

## Graphical abstract

We report an atypical case of Wells syndrome in conjunction with rare eosinophilic disorders such as hypereosinophilic syndrome and eosinophilic granulomatosis with polyangiitis (Churg–Strauss syndrome).

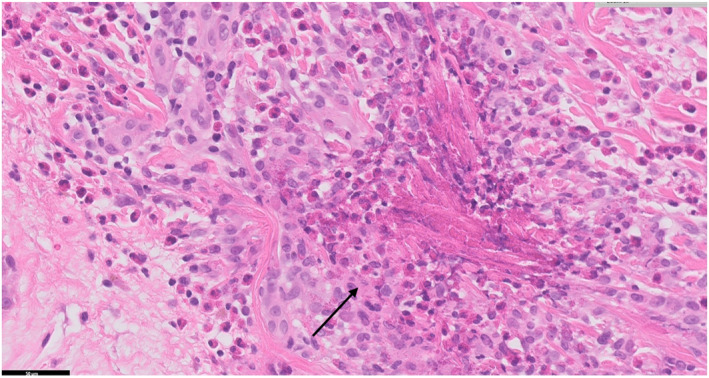

## 1 CLINICAL FINDINGS

A 41‐year‐old Turkish female presented to the clinic with a woody indurated rash and abscess formation on her chest and breasts. She had a history of childhood asthma and a recent diagnosis of hypereosinophilic syndrome (HES) 12 months ago, resulting in end‐organ damage, including myocarditis and pneumonitis.

Her initial presentation included a 4‐month history of cough, chest pain, bone pain, weight loss and night sweats without any haemoptysis. Tuberculosis was initially suspected but negative cultures and eosinophilic rise from 4^9^/L to 11.5^9^/L in a few months prompted further investigations for suspected HES. Laboratory tests were negative for parasite infections, aspergillus precipitants, cytomegalovirus, antinuclear, and antineutrophil cytoplasmic antibodies (ANCA). Raised troponin and ground‐glass changes on CT chest resulted in further cardiac and respiratory investigations: a cardiac MRI showing an ejection fraction of 30% and a myocardial biopsy showing scattered eosinophils and an eosinophilic or giant cell myocarditis to be considered. Bronchoalveolar lavage also showed 26% eosinophils. Further investigations revealed T‐cell proliferation in bone marrow aspiration. *FIP1L1‐PDGFRA* mutation was absent, and a bone marrow biopsy revealed no evidence of malignancy. The patient had been on high‐dose steroids, which initially controlled her blood eosinophilia. However, she developed steroid‐refractory disease, and a 10‐month trial of imatinib was unsuccessful. She remained on a slow‐weaning course of steroids (prednisolone 40 mg when imatinib was discontinued). A few months after her initial presentation, she developed an acneiform eruption on her face and neck. A biopsy suggested eosinophilic folliculitis (Ofuji's disease). At that time, she was on the initial weaning course of steroids (prednisolone 15 mg), and the addition of doxycycline helped resolve the rash.

**FIGURE 1:**
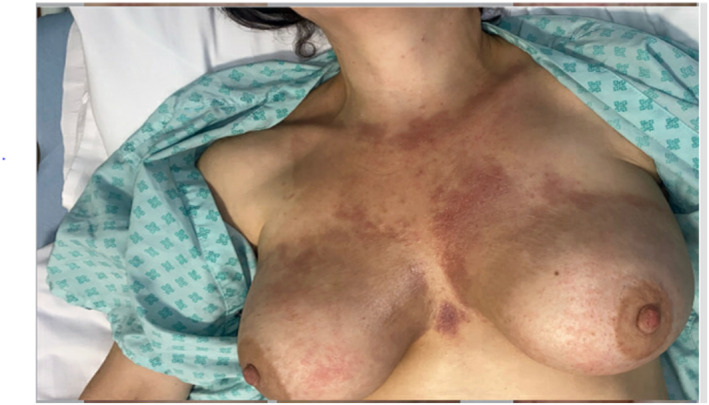
Woody indurated texture of skin over the chest and breasts. Presence of erythema and hyperpigmentation.

**FIGURE 2:**
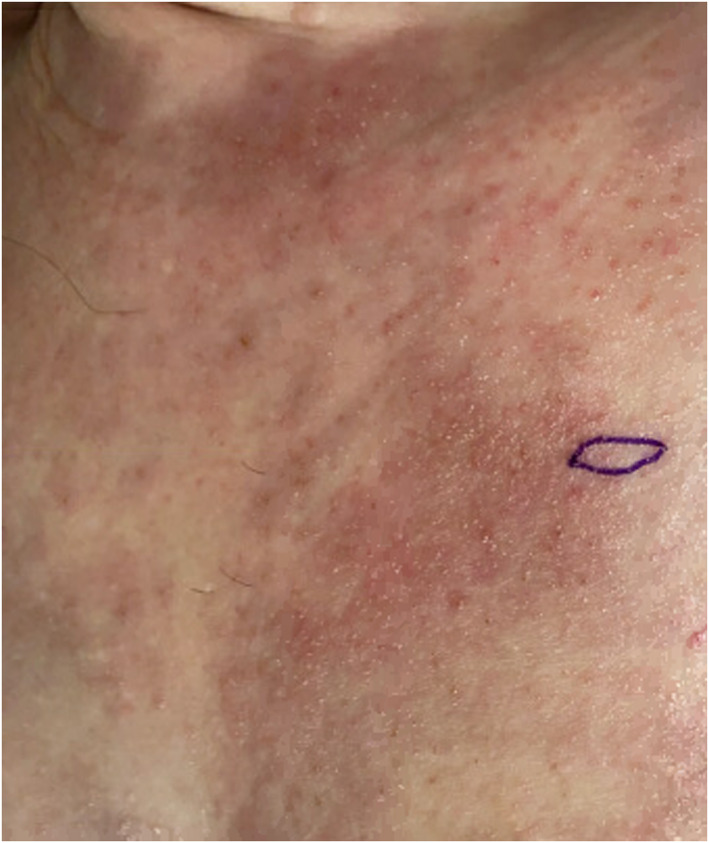
Close‐up view of the rash in Figure 1 that highlights the erythema and hyperpigmentation. Biopsy taken from area circled in purple.

Over a year after her eosinophilic folliculitis diagnosis, the patient developed a new cutaneous presentation characterized by induration and abscesses over the chest and breasts. There was no history of recent infections, arthropod bites, contact with animals or new medications initiated in the preceding months. Examination of the rash revealed erythema and hyperpigmentation (Figures 1 and 2).

An ultrasound of her breasts suggested mastitis. Initial treatment with antibiotics, including a course of oral flucloxacillin (4 g for 1 week), two courses of oral clindamycin (1.2 g for 2 weeks) and co‐trimoxazole (960 mg for 1 month) controlled the inflammation. However, the rash did not respond fully and continued to cause pain, itchiness, and a palpable lump.

The patient was admitted at the time of the rash due to an asthma exacerbation with respiratory compromise and her prednisolone increased from 40 to 60 mg. Spirometry revealed an obstructive pattern. ANCA test was negative.

## 2 HISTOPATHOLOGICAL FINDINGS

An incisional biopsy of the initial acneiform eruption on the right neck, against a background of eosinophilia with myocarditis, lung nodules, and chronic T‐cell deletion on T‐cell receptor gene rearrangement study, suggested eosinophilic pustular folliculitis. Associated features of perforating folliculitis were also noted. Microscopy was negative for bacteria on the Gram stain, and true vasculitic features were absent. Yeast‐like organisms were identified in the stratum corneum without any fungal hyphae.

**FIGURE 3:**
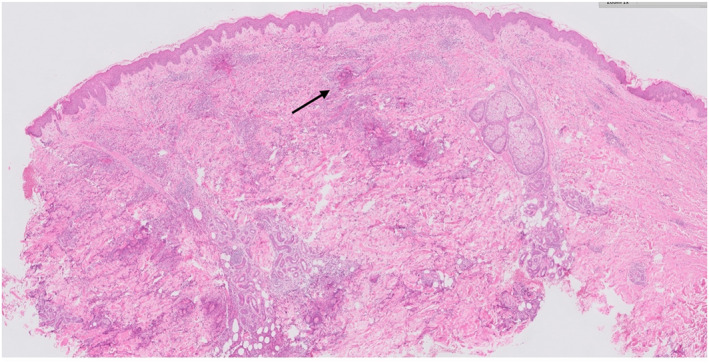
Low power magnification 20 folds (haematoxylin and eosin stain). Demonstrates an infiltrate in the dermis with multiple eosinophilic flame figures. Arrow shows example of flame figure.

**FIGURE 4:**
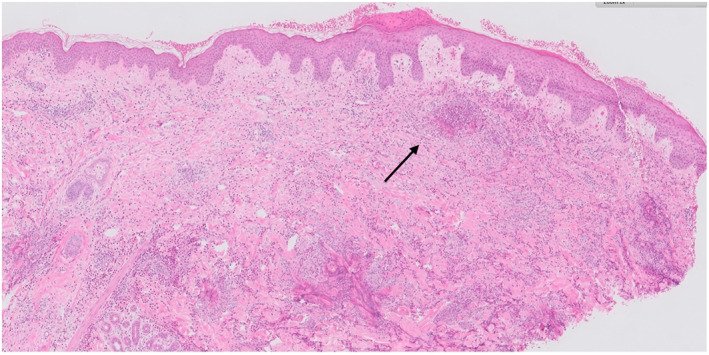
100 folds magnification image of the superficial aspect of the biopsy showing minimal epidermal changes (focal serum in the stratum corneum and mild acanthosis, haematoxylin and eosin stain). Arrow shows example of flame figure.

**FIGURE 5:**
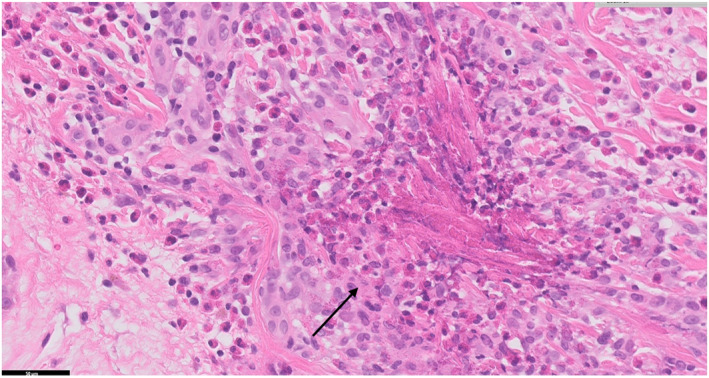
Histopathological appearance of flame figures with surrounding histiocytes 400 folds (haematoxylin and eosin stain). Arrow shows flame figure.

An incisional biopsy of the rash over the breast revealed eosinophilic infiltration of the dermis with flame figures. Vasculocentric inflammation was present, but there were no features of established vasculitis. Some flame figures had formed granulomas with giant cells, appearing older compared to other areas with new flame figures (Figures 3, 4, 5).

## 3 DIAGNOSIS

Eosinophilic cellulitis (Wells syndrome), associated with HES and eosinophilic granulomatosis with polyangiitis (EGPA), previously known as Churg–Strauss syndrome.

## 4 DISCUSSION

To the best of our knowledge, this is the first reported case of eosinophilic cellulitis in a Turkish patient written in English.

The patient presented with various skin manifestations in the context of HES. The initial rash diagnosed as eosinophilic folliculitis responded favourably to weaning steroids and doxycycline. Subsequently, the patient developed a new rash identified as eosinophilic cellulitis, necessitating an increased course of steroids and consideration of other treatment options. Investigation revealed an association with HES and EGPA.

Both eosinophilic folliculitis and eosinophilic cellulitis can be classified as eosinophilic dermatosis, characterized by eosinophilic infiltration of the skin or mucous membranes.^1^ Blood eosinophilia is not a prerequisite for diagnosing these conditions.^1^ Eosinophilic cellulitis has a typical histopathological appearance marked by flame figures, eosinophilic infiltrates, and the absence of vasculitis.^2^ The coexistence of HES and EGPA is remarkably uncommon.

Both cutaneous manifestations and internal organ involvement can present with HES, but skin involvement is generally nonspecific, and the distinct histopathologic findings observed in eosinophilic cellulitis are absent. Histologically, flame figures in HES may appear due to insufficient eosinophil degranulation.^2^

Investigations excluded underlying myeloproliferative or lymphoproliferative disorders. Bone marrow aspiration revealed T cell clonality, whereas bone marrow biopsy was not suggestive of a malignancy. The myeloproliferative variant of HES is typically characterized by a fusion of the uncharacterized *Fip1‐like 1* (*FIPL1*) and platelet‐derived growth factor receptor‐a gene (*PDGFRA*).^2^ High eosinophil counts and the presence of this gene fusion tend to respond favourably to imatinib mesylate.^3^ Imatinib, targeting increased serum concentrations of interleukin‐5 (IL‐5), showed efficacy in HES patients with specific gene mutations, but our patient tested negative for these mutations.^4^

The presence of T cell clonality suggests an increased responsiveness to targeted IL‐5 therapies, given the crucial role of IL‐5 in HES.^2^

Eosinophilic cellulitis can progress to EGPA. Although vasculitis is absent in eosinophilic cellulitis, some cases exhibit both conditions.^5^ In our ANCA‐negative case, the patient had features indicative of EGPA including childhood asthma, previous sinus‐related problems and peripheral blood and tissue eosinophilia with end organ involvement: ground‐glass lung changes and previous pulmonary nodules, eosinophilic cellulitis, myocarditis.^6^, ^7^ However, the 2022 ACR/EULAR classification criteria are challenging to apply due to potential hypereosinophilic asthma mimicking EGPA, given the obstructive pattern on spirometry and the blood eosinophilia.^8^, ^9^ The patient had a history of sinus problems, nasal congestion and occasional nasal blood, pulmonary nodules, and skin granulomas, but was negative for ANCA, antiproteinase 3, and antimyeloperoxidase antibodies. There was no evidence of mononeuritis multiplex or glomerular nephritis.^8^ There were, however, highly suggestive clinical features as mentioned above.^10^ A multidisciplinary approach concluded that diagnosing EGPA and treating it as such would be the most beneficial approach for the patient.

Although initial response to steroids was observed in our patient, as they are utilized in both Eosinophilic cellulitis and EGPA, pulmonary relapses prompted further treatment considerations including anti interleukin 5 targeted therapy. The choice of imatinib was most likely unsuccessful in our patient as the *FIP1L1‐PDGFRA* mutation was absent.^3^

Interleukin‐5 is a key mediator in eosinophil‐related diseases, and medications targeting interleukin‐5 have shown promising results in clinical trials, primarily focusing on asthma but also encompassing HES.^4^

Treatment options include benralizumab (an anti‐interleukin 5Ta receptor antibody), mepolizumab (an anti‐interleukin 5 antibody) and reslizumab (a humanized rat antibody).^4^, ^6^

Benralizumab was used as the final treatment of choice for our patient after comparing efficiency, side effects and cost. Treatment is currently ongoing; however, the treatment is showing initial positive results.

## CONFLICT OF INTEREST STATEMENT

None to declare.

## AUTHOR CONTRIBUTIONS

**Domniki Iatropoulou**: Data curation (lead); project administration (equal); resources (supporting); validation (equal); visualization (lead); writing – original draft (lead); writing – review & editing (equal). **Cathryn Sprenger**: Conceptualization (lead); data curation (equal); resources (equal); supervision (equal); validation (supporting); visualization (equal); writing – original draft (supporting); writing – review & editing (equal). **Richard Griffiths**: Data curation (supporting); resources (lead); software (lead); visualization (equal). **Nilukshi Wijesuriya**: Data curation (supporting); resources (equal). **Bernard Ho**: Conceptualization (lead); data curation (supporting); project administration (lead); resources (supporting); supervision (lead); validation (supporting); visualization (equal); writing – original draft (supporting); writing – review & editing (equal).

## ETHICS STATEMENT

Not applicable.

## PATIENT CONSENT

Written patient consent for publication was obtained.

## Data Availability

The data underlying this article will be shared on reasonable request to the corresponding author.

## References

[1] Marzano AV , Genovese G . Eosinophilic dermatoses: recognition and management. Am J Clin Dermatol. 2020;21(4):525–539. 10.1007/s40257-020-00520-4 32394361

[2] Felix Urman MSPD of DSLRHC . Dermatologic manifestations of hypereosinophilic syndrome clinical presentation. Medscape. 2019. Published online.

[3] Plötz SG , Simon HU , Darsow U , Simon D , Vassina E , Yousefi S , et al. Use of an anti–interleukin‐5 antibody in the hypereosinophilic syndrome with eosinophilic dermatitis. N Engl J Med. 2003;349(24):2334–2339. 10.1056/NEJMoa031261 14668459

[4] Butt NM , Lambert J , Ali S , Beer P , Cross N , Duncombe A , et al. Guideline for the investigation and management of eosinophilia. Br J Haematol. 2017;176(4):553–572. 10.1111/bjh.14488 28112388

[5] Ratzinger G , Zankl J , Zelger B . Wells syndrome and its relationship to Churg‐Strauss syndrome. Int J Dermatol. 2013;52(8):949–954. 10.1111/j.1365-4632.2012.05482.x 23786445

[6] Masi AT , Hunder GG , Lie JT , Michel BA , Bloch DA , Arend WP , et al. The American College of Rheumatology 1990 criteria for the classification of Churg Strauss syndrome (allergic granulomatosis and angiitis). Arthritis Rheum. 2010;33(8):1094–1100. 10.1002/art.1780330806 2202307

[7] Pyo JY , Lee LE , Park YB , Lee SW . Comparison of the 2022 ACR/EULAR classification criteria for antineutrophil cytoplasmic antibody‐associated vasculitis with previous criteria. Yonsei Med J. 2023;64(1):11–17. PMID: 36579374; PMCID: PMC9826961. 10.3349/ymj.2022.0435 36579374 PMC9826961

[8] Robson JC , Grayson PC , Ponte C , Suppiah R , Craven A , Judge A , et al. 2022 American College of Rheumatology/European Alliance of Associations for Rheumatology classification criteria for granulomatosis with polyangiitis. Ann Rheum Dis. 2022;81(3):315–320. Epub 2022 Feb 2. PMID: 35110333. 10.1136/annrheumdis-2021-221795 35110333

[9] Zarka F , Veillette C , Makhzoum JP . A review of primary vasculitis mimickers based on the Chapel Hill Consensus classification. Int J Rheumatol. 2020;2020:8392542. PMID: 32148510; PMCID: PMC7049422. 10.1155/2020/8392542 32148510 PMC7049422

[10] Emmi G , Bettiol A , Gelain E , Bajema IM , Berti A , Burns S , et al. Evidence‐based guideline for the diagnosis and management of eosinophilic granulomatosis with polyangiitis. Nat Rev Rheumatol. 2023;19:378–393. 10.1038/s41584-023-00958-w 37161084

